# A single-stage computer-guided gap arthroplasty with simultaneous patient-specific total joint replacement with a novel flat fossa design: A case report

**DOI:** 10.1016/j.ijscr.2021.01.078

**Published:** 2021-01-22

**Authors:** Mohamed Shawky, Mohamed S. Elbehairy, Mohammed Atef, Khaled Amr

**Affiliations:** a Department of Oral and Maxillofacial Surgery, Faculty of Dentistry, Cairo University, Egypt; b Department of Removable Prosthodontics, Faculty of Oral and Dental Medicine, South Valley University, Qena, Egypt; c Department of Oral and Maxillofacial Surgery, Faculty of Dentistry, Cairo University, Cairo, Egypt

**Keywords:** Patient-specific implant (PSI), CAD/CAM, Titanium, Computer-guided surgery, TMJ, Ankylosis, Polyethelene, Facial asymmetry, Orthognathic

## Abstract

**Introduction and importance:**

The computer-guided approach for the patient-specific TMJ replacement is considered an ultimate reliable option in advanced cases. However, dislocation of the condylar head could happen with the concave fossa design. A flat design was described and used in this case.

**Case presentation:**

A 15 years old male patient with mandibular asymmetry and class IV recurrent ankylosis of the right TMJ received a patient-specific artificial joint with computer-guided gap arthroplasty and orthognathic corrective mandibular surgery for the left side. The fossa component was made entirely flat, and the patient was followed up over the next year.

**Clinical discussion:**

Customized TMJ prosthetic solutions are reliable in advanced cases, especially when facial corrections are indicated; however, the commonly used concave design showed reported dislocations requiring some design modifications as proposed in the presented case.

**Conclusion:**

The flat fossa design of the artificial TMJ secures the same results as the anatomical fossa design without the incidence of dislocation.

## Highlights

• Custom made TMJ total joint replacements are now considered a reliable treatment method.
• Computer guided approach to TMJ reconstruction guarantees the predictability and reliability of the whole surgery.
• Dislocation of the artificial condylar head from the artificial fossa was reported in some cases when the normal concave anatomical fossa design was employed.
• A flattened articular surface of a customized artificial TMJ fossa was employed in this case report.
• The whole computer guided approach allowed for a simultaneous treatment of a recurrent TMJ ankylosis and facial asymmetry through a sagittal split ramus osteotomy of the contralateral side of the mandible.

## 1 Introduction

Ankylosis of the temporomandibular joint (TMJ) is the fusion between the condylar process of the mandible and the glenoid fossa of the temporal bone. It may be fibrous or bony and is manifested as a progressively increasing limitation in mouth opening, a significant decrease in function that affects the dentofacial growth and development if occurred during childhood, resulting in facial asymmetry and malocclusion. It is more common in developing countries due to its prevalence as a complication of improper management of condylar head and sub-condylar fractures in growing children. There are four types of TMJ ankylosis, according to Sawhney 1986 [1]. In type I, there are fibrous adhesions. The bony union is shown in type II on the outer lip of the articular fossa, so along with type III and type IV, they are treated with gap arthroplasty, which refers to the removal of the bony ankylotic mass to create a gap of about 1-1.5 cm with or without the immediate placement of an interpositional substance [[1], [2], [3], [4]].

Placing an interpositional material is advocated to avoid the formation of fibrous adhesions between the bony surfaces after resecting the ankylotic mass, reducing the possibility of re-ankylosis. They include autologous soft tissue substances such as temporalis muscle pedicled flap, fat, skin, auricular cartilage, and fascia lata [5,6]. Alternatively, hard tissue substances such as costochondral (CCG) or sternoclavicular grafts, which are advocated when there is a significant loss of ramus height, in growing children or in bilateral cases to restore the facial height, mandibular form, and function and to benefit from its growth potential [[6], [7], [8], [9]]. On the other hand, some authors do not prefer placing autologous bone after gap arthroplasty; because excess heterotrophic bone already tends to be formed spontaneously in the pathological joint, the early mandibular function may cause some graft mobility, which will jeopardize the process of graft integration and to avoid the morbidity of a new donor site.

Alloplastic alternatives were introduced and were reported to be efficient and safe, and they became the usual means of treatment in developed countries. The TMJ artificial total joint replacements (TJRs) can be ready-made, like the TMJ Concepts Prosthesis (TMJ Concepts Inc., Ventura, CA, USA), formerly Techmedica, and the Biomet Microfixation TMJ Replacement System (Biomet Microfixation, Jacksonville, FL, USA), formerly Lorenz. Alternatively, it can be a customized (patient-specific) joint replacement device or implant [7,[10], [11], [12], [13], [14], [15]].

The customized TJRs have several advantages, including the high accuracy and perfect fit, especially of the artificial fossa component, requiring little - if any - customization is needed of the native bony fossa. It is extremely reliable during operations, especially in recurrent cases and in those with large ankylotic masses where the outline of the normal glenoid fossa was lost. Another advantage is that it permits a simultaneous orthognathic surgical treatment of facial asymmetry cases. Patient-specific TJRs provide predictability and less operating time to gap arthroplasty procedures.

Since the introduction of TMJ TJRs, an anatomic concave fossa design was in use, which mimics the shape of the normal glenoid fossa. Not only has it been incorporated in the ready-made solutions, but even the previous studies on custom made TMJ TJRs had a concave fossa design [16]. With follow up, a significant drawback emerged, which is the dislocation of the artificial condylar head anterior to the fossa component or even lateral dislocation during function. Moreover, this could even occur during extubation or later, especially when the patient experiences nausea and vomiting [17]. So, this raised the suspicion that maybe a flat design of the customized fossa would help avoid this problem.

So in the present study, the flat design of the fossa component of the artificial total joint replacement was introduced in a case with unilateral TMJ ankylosis and facial asymmetry, along with a surgical guide that specifies the locations of the osteotomies and fixation screws for better and predictable placement and fit of the customized TJRs and to control the depth as to avoid injuring the internal maxillary artery.

## 2 Materials and methods

This work has been reported in line with the SCARE 2020 criteria [18], and was registered with the unique identifying number: researchregistry6474.

A 15 years old male patient with an inability to open his mouth came to the outpatient clinic of the department of oral and maxillofacial surgery, faculty of dentistry, Cairo University. The patient didn't suffer from any systemic diseases, he was not following any drug regimens and had no previous dental interventions. There was no relevant family history to the chief complaint. At school he suffered a lowered self-esteem from his facial asymmetry and inability to eat like his fellow colleagues. A history of trauma to the chin due to a fall at the age of five years old was reported by the parents followed by two previous attempts of gap arthroplasty; the first was at the age of nine while the second was at eleven years old. Each of them was followed by a period of normal function, followed by gradual restriction of movements and finally re-ankylosis. Extraoral clinical examination revealed facial asymmetry with a deviated chin to the right side, scarring in the pre-auricular region where an endaural incision was previously attempted twice, the joint space could not be felt by palpation of the right side but a hard continuation of bone from the root of the zygomatic arch to the ramus of the mandible, facial nerve functions were normal at the moment however the parents reported immediate postoperative paresis along the temporal and zygomatic branches after the last operation that resolved two months later.

**Fig. 1:**
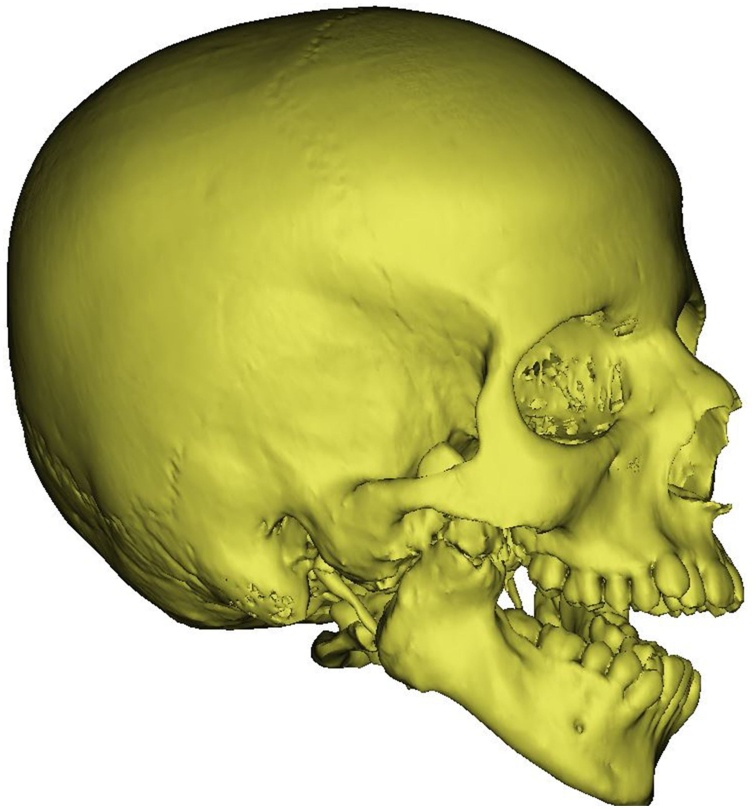
Preoperative 3D volume rendered CT of bone.

Intraoral examination revealed crowding without canting of the maxillary occlusal plane, gingivitis, and multiple carious teeth, which is attributed to the inability to maintain oral hygiene or seek dental care. A preoperative CT (computed tomography) was requested on the full skull to the level of the hyoid bone with 0.6 mm slice thickness and intervals (Siemens CT SOMARIS/10 VA10A, Siemens Shanghai Medical Equipment Ltd.) to find a class IV unilateral (right side) TMJ ankylosis with a fusion of the coronoid process to the zygomatic arch (Fig. 1). The decision to place an artificial TJR simultaneous with orthognathic correction of mandibular deviation was taken after orthodontic consultation and requesting a bone scan scintigraphy to confirm the cessation of growth in the normal (left) condylar growth site. Although, a high uptake in the affected (right) side was found.

The study was approved by the ethics committee of the faculty of dentistry, Cairo University, and was carried out following the rules of the Declaration of Helsinki for medical research involving human subjects. The treatment plan was discussed with the parents, and informed consent was signed. Written informed consent was obtained from the patient for publication of this case report and accompanying images

### 2.1 Preoperative workup

**Fig. 2:**
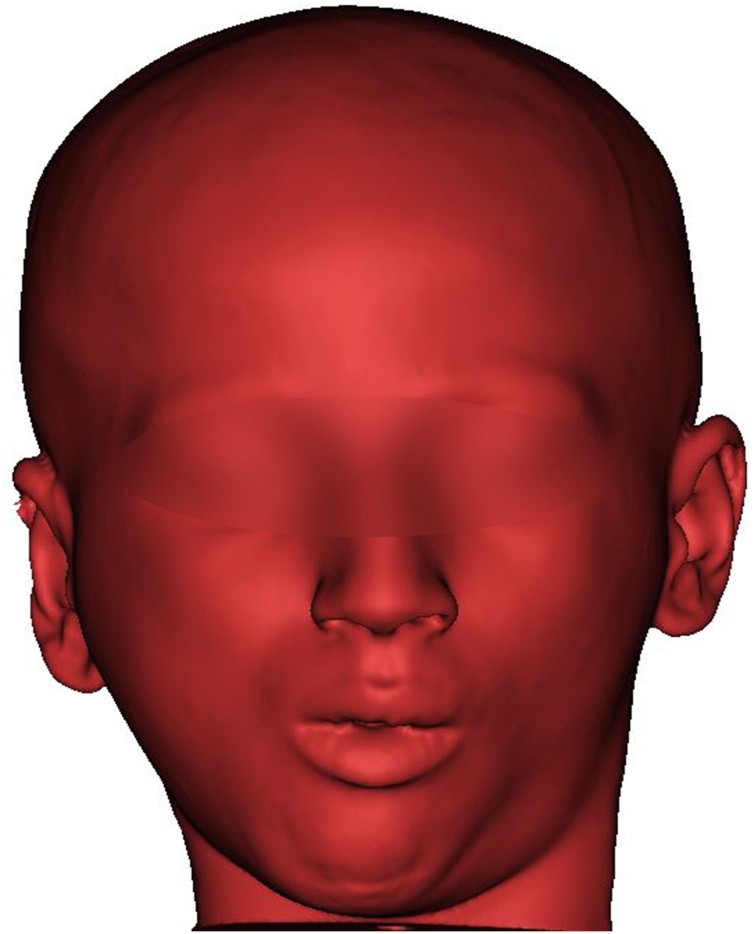
Preoperative 3D volume rendered CT of soft tissue.

**Fig. 3:**
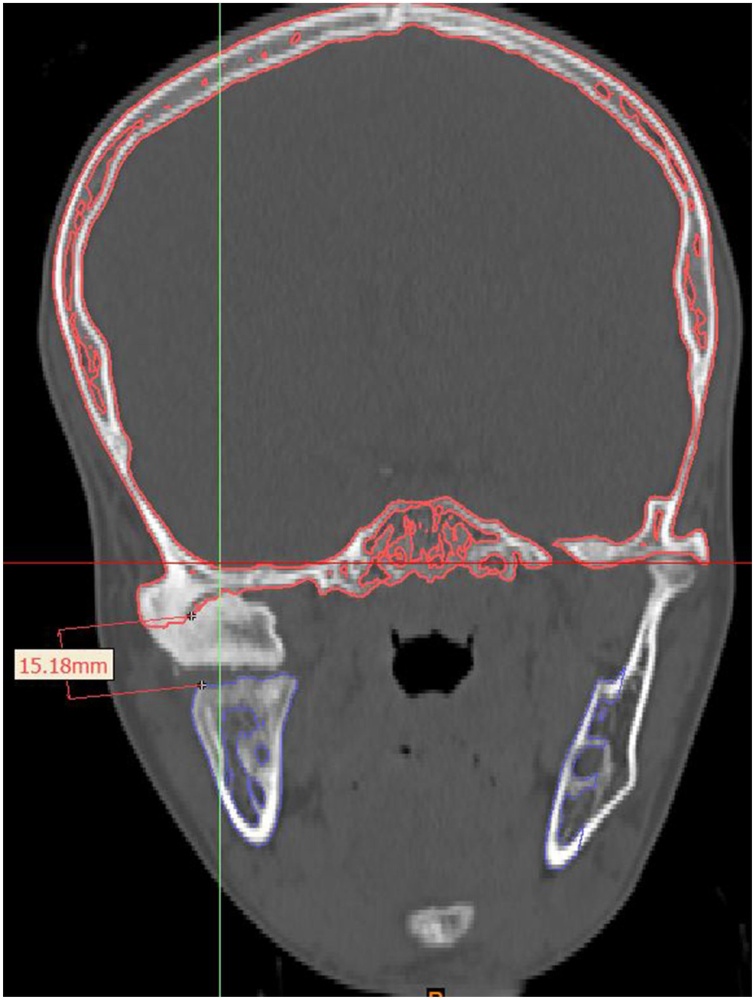
Planning the size of the gap arthroplasty.

**Fig. 4:**
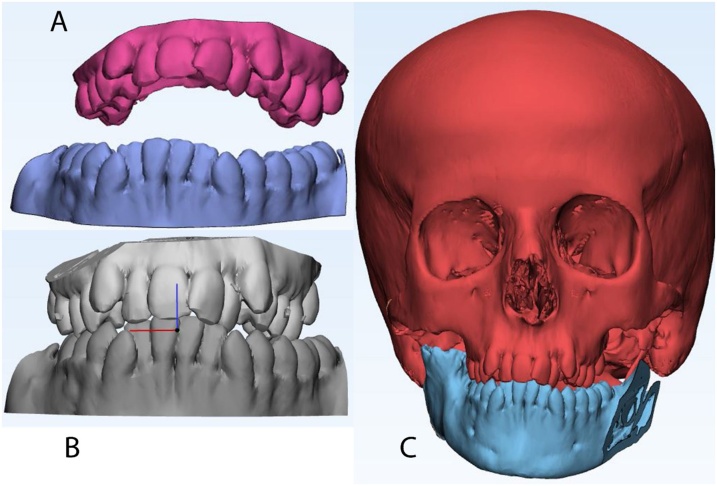
a) The isolated dental arched before 3D printing, b) scanned models of the dental arches after they are brought in occlusion, c) the mandible brought into its intended postoperative occlusion by superimposition.

Mimics 21.0 software (Materialise, Leuven, Belgium) was used to import the DICOM (Digital Imaging and Communications in Medicine) files of the CT scan. Bony and soft tissue masks (Fig. 2) were created and computed into virtual 3D models. The actual extensions of the bony ankylosis were viewed in the three planes and in 3D. The desired resection margins of the bony ankylosis were marked by drawing a plane at the proposed upper and lower margins taking care to keep a gap of 1.5 cm; then, these planes were used to perform a "Cut" resulting in separation of the ankylotic mass (Fig. 3). The remaining skull model was then split to separate the mandible. On the left side, a plane was drawn by which a left side sagittal split of the ramus was done, separating it from the body of the mandible and resembling the future sagittal split osteotomy of the left ramus. The maxillary and mandibular dental arches were also isolated to be converted into casts that were 3D printed. This was done to choose the most stable postoperative occlusion according to which the mandible will be positioned. The final relation was then scanned into STL (Standard Tessellation Language) format, imported, registered, and the distal segment of the mandible was moved virtually in occlusion at the postoperative position (Fig. 4a–c).

**Fig. 5:**
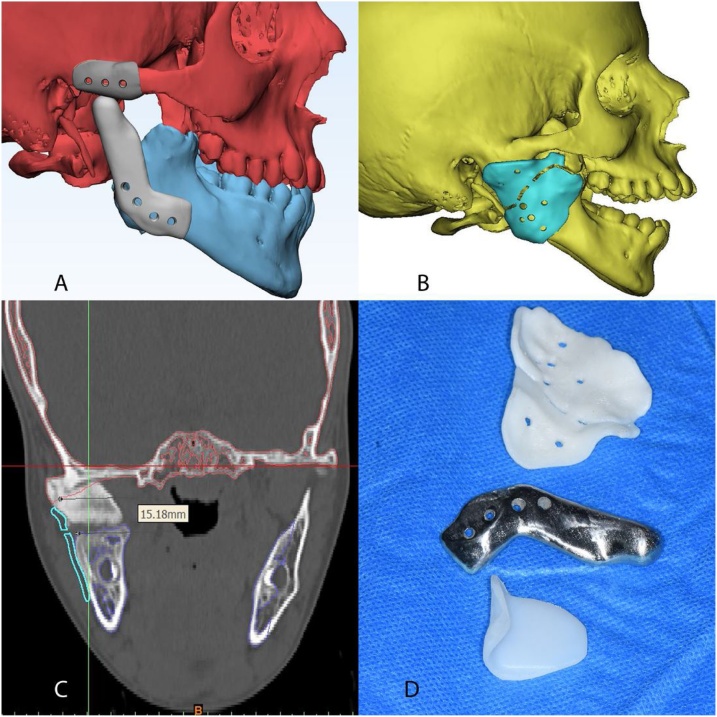
a) Final models of the fossa and condylar components before manufacturing, b) The guide, c) 2D coronal section showing the planned osteotomy locations in the guide, d) Manufactured fossa, condyle and guide.

A midsagittal plane was generated from three midline points; the center of the hypophyseal fossa (Sella turcica), the bony nasion (N), and the anterior nasal spine (ANS) to produce a mirror image of the normal side. Then the virtual models were exported to 3-matic 13.0 software (Materialise, Leuven, Belgium) where the mirrored condyle and fossa from the normal side were used in designing the artificial joint components. The articular eminence was flattened out, and the fossa was extended a little more anterior, eliminating the usual concave design. The medial extension was 2 cm similar to the normal contralateral fossa, and its thickness was 3.5 mm. A fixation arm with three screw holes over the side of the zygomatic arch was designed to hook above the root of the zygomatic arch to validate the accuracy of seating intraoperatively. The condylar component was designed so that the artificial condyle is all rounded with four screw holes in the fixation arm that were located according to the amount of bone to be resected and the relation to the inferior alveolar canal (IAC). The surfaces of the mandible and skull base were then subtracted from the designed parts to ensure optimum adaptation intraoperatively (Fig. 5a).

A 2 mm thick surgical guide was designed so that it has the following functions; locate the upper and lower cuts intraoperatively taking care to include the coronoid process in the resection mass, control the depth of cutting with fissure burs by determining the depth from the guide surface on the CT and to allow the predrilling of the fixation screws of the condylar component. So, in a reverse engineering step, the mandible was brought back to its "home position" while designing the surgical guide so that it can locate the bony cuts and the fixation screws in the current situation (Fig. 5b, c).

### 2.2 Soft tissue simulation

Before exporting the designed parts, a soft tissue simulation of the bony movements' effect was done. Using the previously generated 3D soft tissue model and the moved bony parts in their final postoperative position, the "soft tissue simulation" module in Mimics 21.0 software was used to build a postoperative 3D soft tissue anticipation that was shown to the patient and his parents to appreciate an approximate of the expected change in the facial appearance.

### 2.3 Manufacturing

The designed joint components were exported in STL format, and the condyle component was 3D printed from grade 5 titanium alloy with selective laser melting (SLM) technology. The fossa component was milled from ultra-high molecular weight polyethylene (UHMWPE), while the surgical guide was 3D printed from ABS with (FDM) technology.

The SLM technology is highly accurate but produces a rough surface, so the 3D printed condylar part had to go through a series or smoothening and cleaning steps starting with carbide burs to remove gross irregularities and support remnants, followed by abrasive flap wheels for finishing, then cotton fiber bur impregnated with aluminum oxide, and finally a set of smooth grit polishing wheels with diamond compound for the final polish (Fig. 5d).

### 2.4 Surgical step

The operation was carried out by the authors under general anesthesia, though the preexisting scar on the right side from the previous endaural approaches. After exposing the ankylotic mass, a right retromandibular incision was opened to access the mandibular ramus. The guide was installed in place and fixed with three screws, then the locating holes of the condylar component fixation screws were drilled. A fissure bur was used to mark the upper and lower osteotomies and gradually deepen them in a converging direction until the desired depth was reached guided by a previous depth marking on the shaft of the bur measured from the guide surface.

**Fig. 6:**
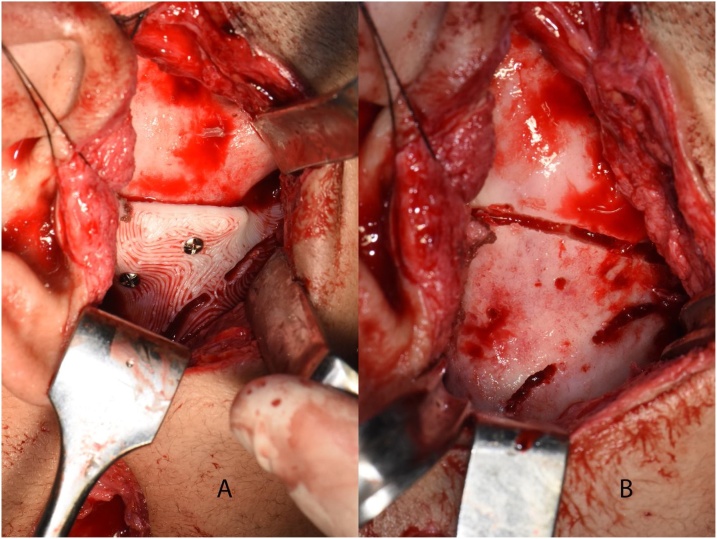
a) The guide installed and fixed in place, b) the upper and lower gap arthroplasty osteotomies marked according to the guide.

**Fig. 7:**
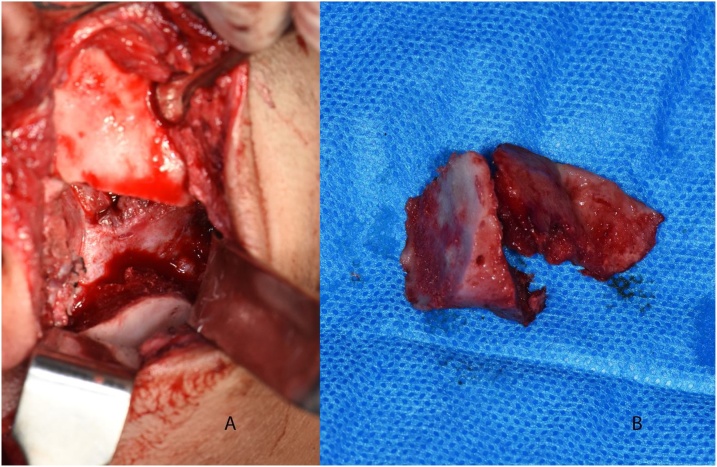
a) The executed gap arthroplasty with intact medial periosteum, b) Resected ankylotic mass.

The guide was then removed, and an Obwegeser chisel was inserted to crack the ankylotic mass loose and remove it (Figs. 6, 7). The mandible was mobilized open, then Erich arch bars were fixed to the upper and lower teeth, a bite block was inserted, and an incision was made on the left ascending ramus. After sub-periosteal exposure, the pterygo-masseteric sling was released, and the osteotomies for the sagittal split were executed; starting with the medial cut using a Lindemann bur; taking care to retract the inferior alveolar nerve (IAN) bundle, then the anterior vertical osteotomy also with a Lindemann bur; taking care to cut through the inferior border. The connecting cut was made with a fissure bur. Spatula chisels were gradually inserted and malleted until the ramus was split without exposure of the IAN. The left wisdom tooth was removed from its crypt.

**Fig. 8:**
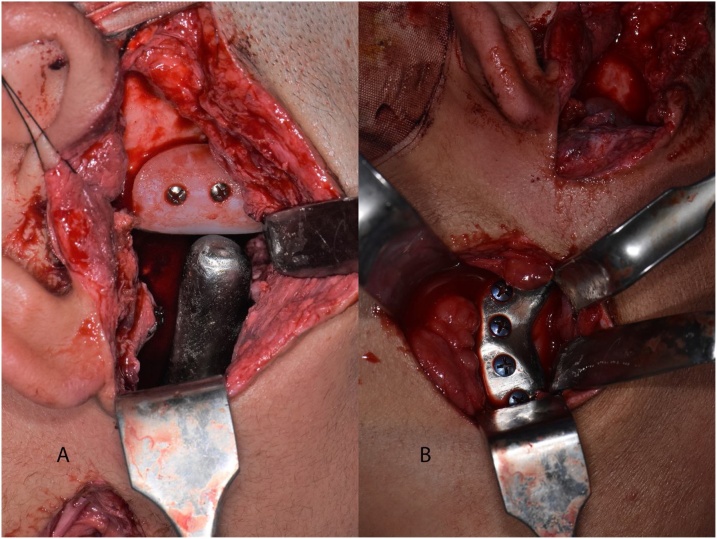
a, b showing the fossa and condyle installed and fixed in place.

The fossa component was inserted; minimal modification in the recipient site was needed before the pre-designed hook verified the full seating. It was fixed with three 2.0 mm mini-screws (Anton Hipp, Fridingen an der Donau, Germany) to the lateral side of the arch, then the condylar component was inserted and fixed with four 2.3 mm screws (Anton Hipp, Fridingen an der Donau, Germany) to the predrilled four screw holes (Fig. 8). Then the mandible was brought manually into the planned occlusion with the maxilla, and intermaxillary fixation was done. After condylar seating of the left proximal segment with gentle upward and backward pressure, it was fixed with a superior border, six holes mini-plate (Anton Hipp, Fridingen an der Donau, Germany) and two, bicortical, 2.0 mm mini-screws (Anton Hipp, Fridingen an der Donau, Germany) placed inferiorly. IMF was released to check occlusion, the seating of the left condyle, and the articulation of the right artificial joint. The intraoral wound was closed with a running 3-0 vicryl (AssuCryl, Assut, Switzerland), both the retromandibular and endaural approaches were closed in layers, and the skin was closed with polypropylene 4-0 (Assut, Switzerland).

**Fig. 9:**
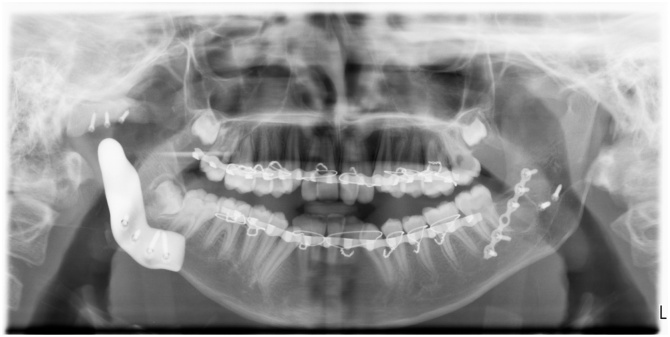
Postoperative panorama.

Postoperative medications involved unictam 1.5 gm IM (Unictam, medical union pharmaceuticals, Cairo, Egypt) twice daily for one week, ketorolac 30 mg IM (Amriya pharm. Alexandria, Egypt) twice daily for five days, and four doses of dexamethasone phosphate 8 mg IM (medical union pharmaceuticals, Cairo, Egypt) on the first 24 hours tapered to the half on the next 24 hours. Oral hygiene instructions with chlorhexidine mouth wash (Orovex, macro group pharmaceuticals, Cairo, Egypt) and soft diet for the first week were stressed, then a gradual return to the usual dietary consistency. Immediate postoperative panorama (Fig. 9) and CT were requested.

Strict postoperative physiotherapy instructions were given. The patient was discharged home on the next day, assessment of the mouth opening, occlusion, pain were carried out in the follow-up visits; after two days, at the end of the first week, then weekly till the end of the first month after that he was recalled monthly for a year. In the follow-up visits, adherence to the physiotherapy instructions was assessed, by observing and documenting the progress in the mouth opening, mandibular functions, and mastication, the ipsilateral facial nerve functions were assessed by asking the patient to raise his eyebrow, close his eyes forcefully, blow his cheeks and move his lower lip.

**Fig. 10:**
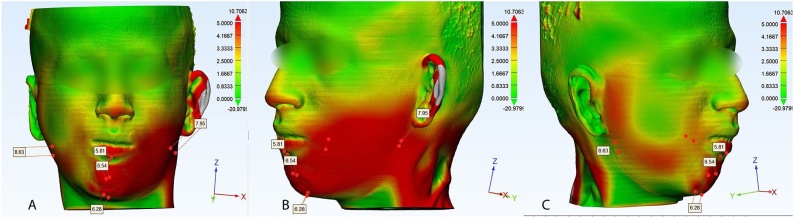
Color map showing the difference between the pre and postoperative soft tissue: A) frontal view, B) Left side, C) right side.

A new CT scan was requested after one year, from which bony and soft tissue 3D models were calculated. The skull models were used as a reference to superimpose the preoperative and postoperative soft tissue models utilizing the 3-matic 13.0 software. Then "Part comparison analysis" was activated to generate a color map showing the soft tissue changes. The following soft tissue landmarks were traced on the preoperative and the postoperative 3D soft tissue models: the soft tissue pogonion, soft tissue menton, soft tissue gonion bilaterally, and the vermillion border of the lower lip. Then, the distance between each point on the preoperative 3D soft tissue model and its corresponding one on the postoperative model was measured (Fig. 10).

**Fig. 11:**
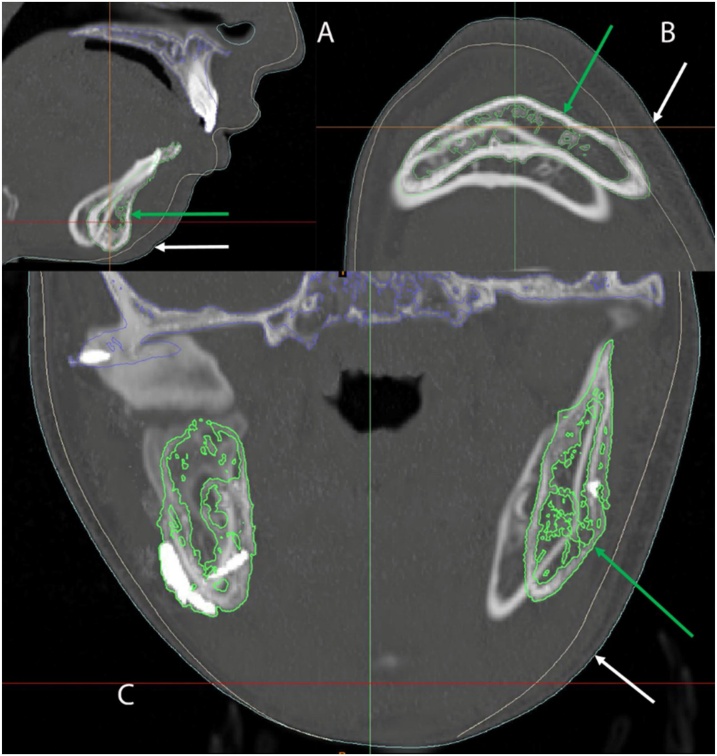
2D CT slices showing the amount of bony and soft tissue correction: A) Sagittal View, B) Axial View, C) Coronal View, *Green arrow representing the postoperative bone, while the white arrow representing the postoperative soft tissue*.

Moreover, on mimics software, the "image registration" module was used to superimpose the entire postoperative project over the preoperative project, using the following reference points; the infraorbital foramina and the superior opening of the incisive canal from the sagittal view, and the optic canals from the axial view. This permitted a 2D appreciation of the difference between the preoperative and postoperative soft tissues by turning on their "contours" on the axial, coronal and sagittal cuts (Fig. 11).

## 3 Results

The computer-guided gap arthroplasty was performed without any intraoperative complications, especially bleeding. According to the plan, the pre-designed artificial fossa and condyle were installed in place without any problems in the desired vertical dimension, and the guided placement of the condylar part according to the predrilled holes was accurate. Sutures were removed at the end of the first week. Paresis affecting all five branches of the facial nerve on the right side was found immediately postoperatively, so B12 complex vitamin was prescribed (Neurobion tab, GlaxoSmithKline, Cairo, Egypt) once every other day. Gradual full recovery was achieved over the next two months. Wound healing went uneventful, and sutures were removed after ten days.

**Fig. 12:**
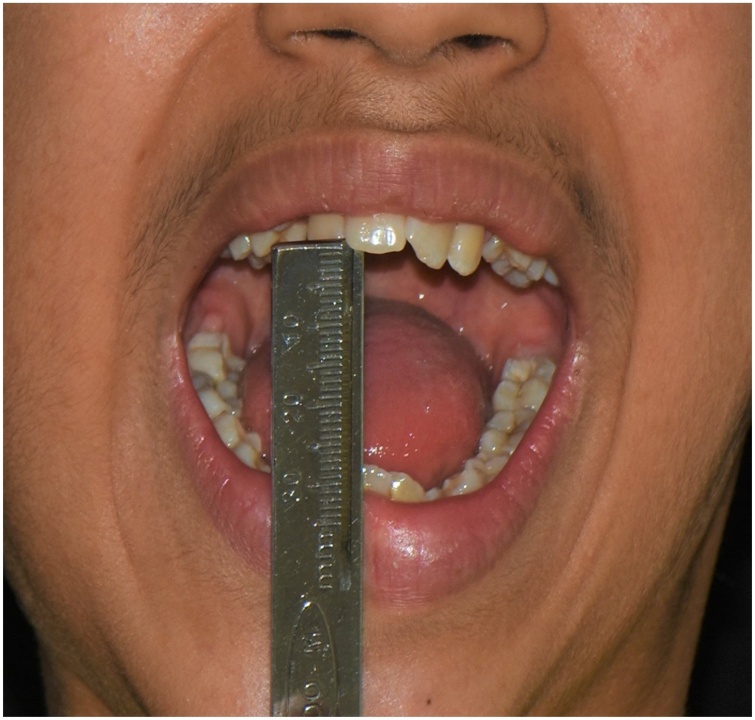
Inter-incisal distance.

The patient reached a 3 cm inter-incisal opening immediately after the operation, and it was maintained over the next year being checked on every follow-up visit (Fig. 12).

The mandibular deviation and shortened right posterior facial height were markedly corrected, guiding elastics were used to preserve the achieved occlusal relationship until the patient started his final orthodontic alignment after three months. After superimposing the 3D model of the mandible from the postoperative CT on the preoperative plan, the screw heads were in the same place as planned. So the guide was concluded to be accurate. Also, through the follow-up visits, no dislocation was reported by the patient or his parents, and normal mandibular functions and dietary habits were reached at the end of the first month.

Comparing the one-year 3D soft tissue model to the preoperative model revealed: a difference of 8.63 mm between the gonion points on the right side, and 7.95 mm for the left side. The amount of change for the lower lip vermillion border was 5.81 mm, while for the pogonion it was 6.54 mm, and the menton was 6.8 mm. Moreover, the color map generated from part comparison analysis specified the regions where the difference between the preoperative and postoperative situations was more than 3 mm with the red color as shown in (Fig. 10).

## 4 Discussion

In the presented case, the patient suffered from a second recurrence of the right TMJ ankylosis with a greater extension and fusion of the coronoid process to the zygomatic arch, at the same time, there was a significant loss of the right ramus height from the previous two-gap arthroplasty operations with chin deviation to the affected side. So a customized TMJ TJR was selected, being widely accepted to be functionally predictable and reliable. Its use has extended to involve; inflammatory joint disorders that are refractory to treatment, fibrous or bony ankylosis, especially in recurrent cases and previously failed TMJ grafting procedures. Moreover, the customization of the artificial joint replacement for every single case saves time, allows for predictable simultaneous orthognathic maxillo-mandibular corrections [7,12], and all that is guaranteed to be highly accurate, especially when the procedure is totally computer-guided as presented.

A customized artificial TMJ TJR of the right side with simultaneous unilateral sagittal split ramus osteotomy was selected as a definitive treatment to resect the bony ankylosis, correct the mandibular deviation and reconstruct the right TMJ. However, the usual treatment protocol in children with type IV TMJ ankylosis would be an extensive removal of the ankylotic mass followed by coronoidectomy of the affected side, lining the joint space with a temporalis flap and reconstructing the ramus - condyle component either with a CCG or a distraction osteogenesis device [19] in order to preserve the normal facial growth and development. However, since it is already the second recurrence, placing an autogenous bone graft in a region in which heterotrophic bone is spontaneously formed was not preferred [10,20].

So for the placement of alloplastic interpositional material, we had to confirm that the mandibular growth has stopped on the left side with a bone scan scintigraphy first [21], which reinforced the decision to avoid a CCG because of the reported unexpected overgrowth [22,23]. After that, orthodontic consultation was done to agree on the occlusion that will be obtained after the left side sagittal split for correction of the mandibular deviation, after which the patient will start his orthodontic treatment. This was done using a 3D printed dental arches from the original CT, which were then scanned to transfer the desired occlusal relationship back to the software.

The 3D printed models were generated from the CT scan. The complete inability to open the jaw didn't allow impressions or even intraoral optical scans to be obtained. This led to a less optimum accuracy in the generated dental anatomy of the teeth in the 3D printed arches than if stone models or optical scans were used, but this was the most feasible option to select and digitize the postoperative occlusion given the current condition of the patient (prediction

The use of a surgical guide reduced the surgical time and improved both safety and accuracy [24]. It was reported that when an excess bone is removed during ankylotic mass resection, it affects the final adaptation and fit of the customized artificial joint components. So not only was the guide designed to specify the amount of bone removal, but also to localize the positions of the fixation screws of the condylar component for accurate placement. The thickness of the guide was 2 mm in order to provide it with sufficient strength to withstand manipulation during the operation without breakage; it was printed with the ABS material using the fused deposition modeling technology, which is known to have reasonable accuracy with regard to its cost [25]. The superior edge of the guide marked the upper cut while a slit placed 1.5 cm inferiorly marked the lower cut taking care to perform them intraoperatively in a converging direction, especially the upper cut to protect the middle cranial fossa and to facilitate its removal.

No cleavage plane was identified in the CT, so while designing the guide, the upper cut was located away from the floor of the middle cranial fossa by 7 mm. However, since the normal fossa on the contralateral side was measured to be 5 mm away, these additional 2 mm were meant to be removed intraoperatively with a rose head bur until the artificial fossa fits in place guided by the hook in its lateral surface so that finally the artificial fossa is 5 mm away from the floor of the middle cranial fossa similar to the normal side. This was believed to be safer to avoid inadvertent perforation into the middle cranial fossa if any intraoperative modifications were needed. The used fissure burs were marked to control the depth of cutting relative to the guide surface

The fossa was manufactured from UHMWPE with a 3.5 mm thickness as it is characterized by high tensile strength, ductility, and high wear resistance [26]. Its medial extension was 2 cm like the normal contralateral one, and it was completely flat in order to permit free translation without dislocation, which was apparent through the follow-up period; the patient had normal jaw movements and 30 mm interincisal opening without dislocation of the artificial condyle throughout the one year follow up or during extubation. This also proves that a flattened design eliminated the need to place the articulation of the artificial joint at a lower level than the normal, which was believed to reduce the translation [27,28], which would cause dislocation if it occurred [17]. On the contrary, it permitted a free and convenient range of motion as long as it is a unilateral case where the normal contralateral condyle controls the range of motion, so the artificial condyle will not exceed the normal limits but will move freely in the normal range without complications.

The condylar component was designed to be ball rounded and smaller in size than the natural biconvex condylar anatomy, and also, the condyle was placed a little medial to its normal position in order not to be felt outside or produce an over contoured profile. The fixation arm was designed to be adapted on the posterior and inferior borders so that the fixation screws are away from the IAN and to permit the reattachment of the masseter muscle to the ramus. It was 3D printed totally from titanium, although some authors reported that it could be manufactured from Co-Cr-Mo (Cobalt-Chrome-Molybdenum) as a condylar part and titanium as a mandibular part [12,29]. However, titanium can be used solely according to others [27] as it is known for its biocompatibility, and also it has been long used in orthopedic replacements [30].

Grade 5 titanium powder was used to 3D print the condylar component using Selective laser melting (SLM) technology. It is a type of additive manufacturing that was known to be accurate but to generate a rough surface [30,31]. So, a smoothening and cleaning protocol was followed to generate a smooth and polished surface to avoid soft tissue irritation. Carbide burs were first used to remove gross irregularities and support remnants followed by using abrasive flap wheels for finishing then cotton fiber bur impregnated with aluminum oxide and finally a set of smooth grit polishing wheels with diamond compound for the final polish.

Intraoperatively, the followed protocol in the sequence was; bony ankylosis removal with ipsilateral coronoidectomy, contralateral sagittal split osteotomy, IMF then artificial TMJ placement, and rigid fixation of the contralateral ramus after condylar seating. According to Kaban et al. [19,32], there is no need to perform coronoidectomy in the contralateral side if an interincisal opening of more than 35 mm was obtained, which was the case intraoperatively, so coronoidectomy was not done on the normal side. Also, in order not to complicate the planned sagittal split of the contralateral ramus. So, to manage the healthy side's shortened temporalis muscle from the long-standing and recurrent ankylosis, aggressive postoperative physiotherapy instructions were given.

The guided operation and customization of the treatment were beneficial in reducing surgical time, avoiding other donor site morbidity, improve the predictability and safety of the operation. It is known that the TMJ is anatomically related to the internal maxillary artery medially, and avoiding its injury cannot be guaranteed, especially while working in the medial aspect of the ankylotic mass even with proper field exposure. Its first two parts lie just medial to the condylar neck, making it susceptible to injury, especially in large ankylotic masses. It is the larger terminal branch of the external carotid artery; it causes severe blood loss requiring an immediate blood transfusion. Achieving hemostasis is difficult due to the inaccessibility and poor visualization. Some solutions have been proposed, including preoperative selective embolization of the internal maxillary artery [33] or intraoperative emergency ligation of the external carotid artery [34,35], but it is preferred to avoid this whenever possible. The depth of cutting was predetermined from the guide surface, which was believed to guard against accidental injury of the internal maxillary artery. This was evident by the intact medial periosteum that appeared immediately after the removal of the ankylotic mass.

The patient was satisfied with the regained mandibular functions and corrected facial appearance, appreciating a better life with higher self-confidence and esteem.

## 5 Conclusion

In complicated TMJ ankylosis cases, the computer-guided customized approach is needed to enhance the predictability of the surgical intervention, the preoperative patient education, and offer a more accurate postoperative assessment of the entire intervention. The flat fossa design of the artificial TMJ secures the same results as the anatomical fossa design without the incidence of dislocation.

## Declaration of Competing Interest

The authors declare that there is no conflict of interest within the submitted work

## Funding

There were no sources of funding.

## Ethical Approval

The study was approved by the ethics committee of the faculty of dentistry, Cairo University.

## Consent

The study was approved by the ethics committee of the faculty of dentistry, Cairo University.

The treatment plan was discussed with the parents, and informed consent was signed.

Written informed consent was obtained from the patient for publication of this case report and accompanying images. A copy of the written consent is available for review by the Editor-in-Chief of this journal on request.

All that was mentioned within the submitted manuscript.

## Author contribution

Mohamed Shawky (MS), Mohamed S. Elbehairy (MB), Mohammed Atef (MA), Khaled Amr (KA)

1) Study design, data collection, analysis, and interpretation: (MA),(MS), (MB), and (KA).
2) Manuscript drafting/proofreading: (KA), (MA) and (MB) with support from (MS).
3) Critical revision: (KA), (MA), and (MS) with support from (MB).
4) Providing general advice on the study: (MS), (MA) and (KA) with support from (MB).

## Registration of Research Studies

1. Name of the registry: https://www.researchregistry.com/.
2. Unique identifying number or registration ID: researchregistry6474.
3. Hyperlink to your specific registration (must be publicly accessible and will be checked): https://www.researchregistry.com/register-now#home/registrationdetails/6006c48398eb25001e077427/.

## Guarantor

Mohamed Shawky.

## Provenance and peer review

Not commissioned, externally peer-reviewed.
